# Arbuscular mycorrhizal fungal interactions bridge the support of root‐associated microbiota for slope multifunctionality in an erosion‐prone ecosystem

**DOI:** 10.1002/imt2.187

**Published:** 2024-03-25

**Authors:** Tianyi Qiu, Josep Peñuelas, Yinglong Chen, Jordi Sardans, Jialuo Yu, Zhiyuan Xu, Qingliang Cui, Ji Liu, Yongxing Cui, Shuling Zhao, Jing Chen, Yunqiang Wang, Linchuan Fang

**Affiliations:** ^1^ State Key Laboratory of Soil Erosion and Dryland Farming on the Loess Plateau Northwest A&F University Yangling China; ^2^ College of Natural Resources and Environment Northwest A&F University Yangling China; ^3^ Key Laboratory of Green Utilization of Critical Non‐metallic Mineral Resources, Ministry of Education Wuhan University of Technology Wuhan China; ^4^ Consejo Superior de Investigaciones Científicas Global Ecology Unit Centre de Recerca Ecològica i Aplicacions Forestals‐Consejo Superior de Investigaciones Científicas‐Universitat Autònoma de Barcelona Bellaterra Spain; ^5^ Centre de Recerca Ecològica i Aplicacions Forestals Cerdanyola del Vallès Catalonia Spain; ^6^ School of Agriculture and Environment, Institute of Agriculture The University of Western Australia Perth Western Australia Australia; ^7^ Key Laboratory of Ecosystem Network Observation and Modeling, Institute of Geographic Sciences and Natural Resources Research Chinese Academy of Sciences Beijing China; ^8^ Institute of Soil and Water Conservation Chinese Academy of Sciences and Ministry of Water Resources Yangling China; ^9^ Hubei Province Key Laboratory for Geographical Process Analysis and Simulation Central China Normal University Wuhan China; ^10^ Institute of Biology Freie Universität Berlin Berlin Germany; ^11^ Department of Cardiology Renmin Hospital of Wuhan University Wuhan China; ^12^ Chinese Academy of Sciences Center for Excellence in Quaternary Science and Global Change Chinese Academy of Sciences Xi'an China

**Keywords:** arbuscular mycorrhizal fungi, degraded ecosystems, multifunctionality, root‐associated microbiota, slope, soil erosion

## Abstract

The role of diverse soil microbiota in restoring erosion‐induced degraded lands is well recognized. Yet, the facilitative interactions among symbiotic arbuscular mycorrhizal (AM) fungi, rhizobia, and heterotrophic bacteria, which underpin multiple functions in eroded ecosystems, remain unclear. Here, we utilized quantitative microbiota profiling and ecological network analyses to explore the interplay between the diversity and biotic associations of root‐associated microbiota and multifunctionality across an eroded slope of a *Robinia pseudoacacia* plantation on the Loess Plateau. We found explicit variations in slope multifunctionality across different slope positions, associated with shifts in limiting resources, including soil phosphorus (P) and moisture. To cope with P limitation, AM fungi were recruited by *R. pseudoacacia*, assuming pivotal roles as keystones and connectors within cross‐kingdom networks. Furthermore, AM fungi facilitated the assembly and composition of bacterial and rhizobial communities, collectively driving slope multifunctionality. The symbiotic association among *R. pseudoacacia*, AM fungi, and rhizobia promoted slope multifunctionality through enhanced decomposition of recalcitrant compounds, improved P mineralization potential, and optimized microbial metabolism. Overall, our findings highlight the crucial role of AM fungal‐centered microbiota associated with *R. pseudoacacia* in functional delivery within eroded landscapes, providing valuable insights for the sustainable restoration of degraded ecosystems in erosion‐prone regions.

## Highlights

- *Robinia pseudoacacia* strategically recruited AM fungi to cope with phosphorus limitation.
- AM fungi interacted with the assembly and composition of bacteria and rhizobia.
- AM fungal‐centered underground network supported slope multifunctionality.
- The *R. pseudoacacia*–AM fungi–rhizobia association restores eroded ecosystems.

## Graphical abstract

*Robinia pseudoacacia* strategically recruited arbuscular mycorrhizal (AM) fungi to cope with phosphorus limitation. AM fungi interacted with the assembly and composition of bacteria and rhizobia. AM fungal‐centered underground network supported slope multifunctionality. The *R. pseudoacacia*–AM fungi–rhizobia association restores eroded ecosystems.

## INTRODUCTION

Soil microbiota plays a pivotal role in restoring degraded ecosystems by executing an array of essential functions, such as carbon (C) storage, nutrient cycling, organic matter decomposition, and primary production, especially in plantations facing severe soil erosion [1]. As the major biome for restoration, plantations foster a rich biodiversity of soil microbiota by providing conducive habitats (e.g., the rhizosphere), thus supporting high‐level resistance and resilience to soil erosion [1, 2]. Such capabilities largely hinge on the intricate biological interactions within and among plants and microbiota in the rhizosphere, notably involving fungal and bacterial symbiotic associations with plants [3, 4, 5]. However, the extent to which diverse root‐associated microbes and their interactions drive multiple basic ecosystem functions, known as multifunctionality, in plantations remains virtually unknown.

*Robinia pseudoacacia* stands out as a priority species for rehabilitating degraded ecosystems, thanks to its beneficial symbiosis with nitrogen (N)‐fixing rhizobia and high erosion tolerance [6]. In addition to rhizobial symbiosis, mutualisms with arbuscular mycorrhizal (AM) fungi enhance the acquisition capacity of limited nutrients, especially phosphorus (P), by legume trees [7, 8]. This mycorrhizal association potentially interacts with symbiotic N_2_‐fixers (rhizobia), exerting synergistic effects on plant performance by modifying the rhizosphere microbiota [9, 10]. Woody legumes and their root‐associated microbiota have also been reported to boost additional nutrient cycling and organic matter decomposition via mineral weathering that can improve their access to available nutrients in nutrient‐limited soils [11]. Nevertheless, there is evidence that rhizobia inoculation may suppress the subsequent colonization of AM fungi, and vice versa [12]. These findings imply the cooperation or competition between AM fungi and rhizobia established along their co‐evolution [10], but to date, our understanding of the interactions among these symbiotic guilds and other microbiota in the rhizosphere of *R. pseudoacacia* is still limited.

In *R. pseudoacacia* plantations, various abiotic factors markedly influence the diversity or biological interactions of different microbial kingdoms and thereby the delivery of ecosystem functions [13, 14]. The slope is a key landscape feature of *R. pseudoacacia* plantations and also the basic unit to evaluate the impact of landscape position in eroded ecosystems [15]. Through the differentiation of vegetation and the redistribution of soil resources, slope position can have a strong impact on soil N mineralization, biological P utilization, and the biodiversity–ecosystem functioning relationship [16, 17, 18]. Meanwhile, low P availability is prevalent in *R. pseudoacacia* plantations from erosion‐prone areas, limiting plant production and microbial‐driven biogeochemical cycles on the slope [16, 19, 20]. These highly variable abiotic factors are likely to influence the co‐evolution of symbiotic guilds and rhizosphere microbiota, as well as the associated multifunctionality [21]. Unfortunately, existing knowledge about microbial diversity and functionality shifts along slopes is fragmented, with the focus limited to a single microbial kingdom or ecosystem function [13, 22]. In addition, the commonly adopted relative abundance profiling of microbiota fails to compare cross‐kingdom biotic associations between samples [10]. These gaps impede our insights into the underlying mechanisms of natural restoration and the development of relevant management strategies.

Ecological networks serve as useful tools for comprehending complex biological interactions and can disentangle the roles of keystone microbial taxa in community assembly and ecosystem functioning [23, 24]. In eroded landscapes, dominant keystone taxa in network clusters have been identified as the engine to support multifunctionality and mitigate erosion impacts [25, 26]. Similarly, both microbial network complexity and interactions within and among fungal and bacterial kingdoms are critical for maintaining multifunctionality during natural restoration processes [27, 28]. A quantitative profiling method has recently been utilized to uncover new features in the assembly of rhizosphere microbiota [29]. This method is expected to shed light on the symbiosis performance of AM fungi and rhizobia, as well as their interactions [10]. Given its significance to the restoration of eroded ecosystems, a quantitative analysis of network‐based interactions among *R. pseudoacacia* root‐associated microbiota and their linkages with multifunctionality becomes essential.

In this study, we investigated the diversity and cross‐kingdom biotic associations of root‐associated microbiota (i.e., AM fungi, rhizobia, and bacteria) on a typical slope of an *R. pseudoacacia* plantation on the Loess Plateau, China. The Loess Plateau is a traditionally erosion‐induced degraded region but has been partially restored since the initiation of the Grain‐for‐Green Project [30]. To ascertain the support of bacterial and symbiotic kingdoms for ecosystem restoration, we explored their relationship with ecosystem functions across the slope, including individual functions, multidimensional functioning, and multifunctionality. These measurements of slope multifunctionality allow us to generate an integrative recognition of functional delivery at a high level of organization [31]. Furthermore, we quantified the relative contributions and influence pathways of abiotic and biotic factors to understand the mechanistic linkages between microbial associations and slope multifunctionality. Our hypotheses posited that (1) all facets of multifunctionality would be driven by and dependent on the diversity of root‐associated microbiota, among which AM fungi were the foremost, given their proficiency in alleviating P limitation for *R. pseudoacacia*; (2) cross‐kingdom networks would be more connected than single‐kingdom networks, with AM fungi serving as the central figure due to their fundamental role in niche partitioning and interactions with other microbial assemblies [32]; and (3) regardless of the resource distribution along the slope, AM fungal interactions would robustly support multifunctionality, particularly the mutualism with rhizobia that benefits *R. pseudoacacia* by supplying N and P synergistically.

## RESULTS

### Changes in multiple facets of ecosystem functioning along the slope

**Figure 1:**
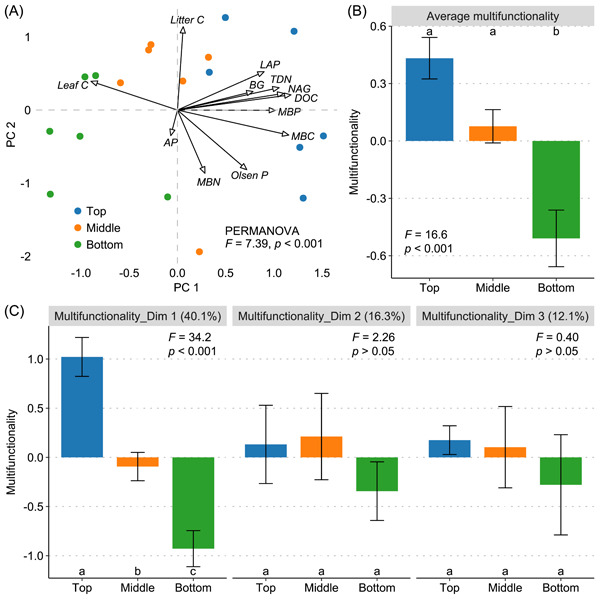
Distribution of multi‐faceted multifunctionality on the slope. (A) Biplot showing key dimensions of 12 individual ecosystem functions from principal component analysis. Different colors represent different slope positions (top, middle, and bottom). Statistical significance of overall functioning among slope positions was tested by permutational multivariate analysis of variance based on the Bray–Curtis distance. (B) Average multifunctionality and (C) multidimensional functioning (eigenvalues for dim 1–3) at three slope positions. Numbers in faceted brackets represent the explained variances for corresponding dimensions. Different lowercase letters indicate significant differences (*p* < 0.05) among slope positions. Values are the means ± standard error (*n* = 6). AP, alkaline phosphatase; BG, *β*‐glucosidase; DOC, dissolved organic carbon; LAP, leucine aminopeptidase; leaf C, leaf carbon content; litter C, litter carbon content; MBC, microbial biomass carbon; MBN, microbial biomass nitrogen; MBP, microbial biomass phosphorus; NAG, *N*‐acetylglucosaminidase; Olsen‐P, available phosphorus; PC 1, the first axis of principal component analysis; PC 2, the second axis of principal component analysis; TDN, total dissolved nitrogen.

Slope position significantly impacted multiple ecosystem functions and thereafter the average multifunctionality, as demonstrated by the permutational multivariate analysis of variance (PERMANOVA; *F* = 7.39, *p* < 0.001; Figure 1A,B and Table S1). Notably, several functions linked to soil and associated with key services experienced a decline down the slope, including microbially driven C pools—dissolved organic carbon (DOC) and microbial biomass carbon (MBC), nutrient cycling—microbial biomass phosphorus (MBP), and organic matter decomposition—*N*‐acetylglucosaminidase (NAG) and leucine aminopeptidase (LAP). In contrast, a relatively higher leaf C content (plant production) was observed at the middle and bottom positions (*p* < 0.01).

Three pivotal dimensions of slope multifunctionality were identified using principal component analysis (PCA), jointly capturing 68.4% of the spatial variation in ecosystem functions (Figure 1A,C). Among them, the first dimension (PC 1), which explained 40.1% of the variance and was dominated by microbially driven C pools and organic matter decomposition, showed a distribution similar to that of the average multifunctionality. Consequently, the negative change of PC 1 was also correlated with leaf C content.

### Root‐associated microbiota and its contribution to slope multifunctionality

**Figure 2:**
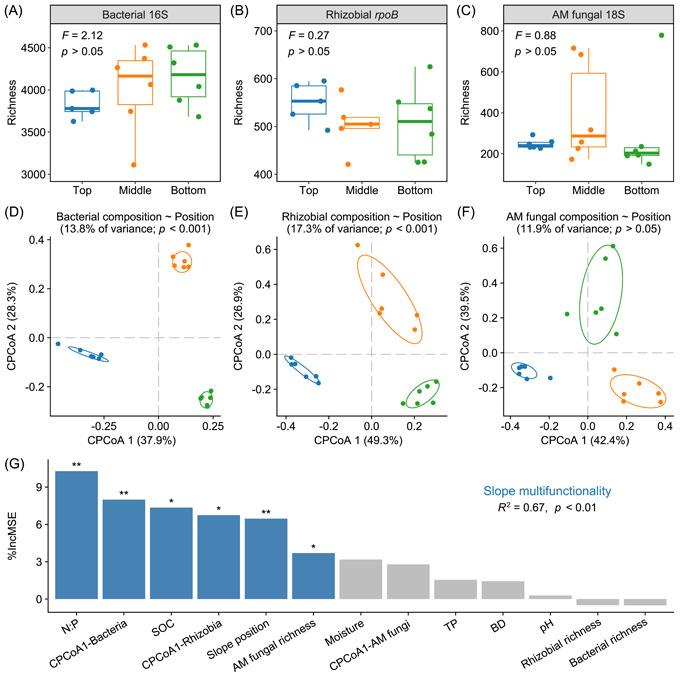
Diversity and composition of root‐associated microbiota driving slope multifunctionality. Richness of bacteria (A), rhizobia (B), and arbuscular mycorrhizal (AM) fungi (C) estimated using 16S *rRNA*, *rpoB*, and 18S *rRNA* genes, respectively (*n* = 6). Statistical significance among slope positions was analyzed via linear mixed‐effects models. Differentiation in the composition of bacterial (D), rhizobial (E), and AM fungal communities (F) based on the Bray–Curtis distance constrained by slope position. Ellipses denote the 95% confidence intervals. Different colors represent different slope positions (top, middle, and bottom). Numbers in faceted brackets indicate the explained variances and significances for individual taxa. (G) Potential drivers of slope multifunctionality. Percentage increases in mean squared error (%IncMSE) were used to evaluate the relative importance of drivers. Asterisks denote the significant contribution to slope multifunctionality. *p < 0.05; **p < 0.01. BD, bulk density; CPCoA 1, the first axis of constrained principal coordinate analysis; CPCoA 2, the second axis of constrained principal coordinate analysis; N:P, soil nitrogen‐to‐phosphorus ratio; SOC, soil organic carbon; TP, total phosphorus.

Despite the large heterogeneity within each position, there was no statistically significant difference in the richness of root‐associated microbiota among slope positions, encompassing bacteria, rhizobia, and AM fungi (Figure 2A–C). However, the biplot of constrained principal coordinate analysis (CPCoA) distinctly illustrated the significant influence of slope position on the composition of bacterial and rhizobial communities (though not present in AM fungal composition), with explained variations of 13.8% and 17.3%, respectively (*p* < 0.001; Figure 2D–F). In terms of these community compositions, the main bacterial order across the slope was Rhizobiales (10.1%–11.3%), while the dominant genus of rhizobia was *Bradyrhizobium* (29.2–39.4%; Figure S1).

Random forest analysis identified bacterial and rhizobial compositions (the first axis of CPCoA) and AM fungal richness as significant predictors for slope multifunctionality (Figure 2G). Specifically, AM fungal richness strongly supported multifunctionality, primarily due to its positive effects on DOC, MBP, and *β*‐glucosidase (BG) (Figure S2). Bacterial and rhizobial compositions exerted more substantial regulatory effects on multifunctionality as observed by their stronger associations with microbially driven C pools (e.g., MBC) and organic matter decomposition (e.g., NAG and LAP). Additionally, soil nitrogen‐to‐phosphorus (N:P) and soil organic carbon (SOC), which decreased from the top to the bottom position, emerged as crucial abiotic factors influencing slope multifunctionality (Figure 2G and Table S2).

### Microbial co‐occurrence patterns and biotic associations of different kingdoms

**Figure 3:**
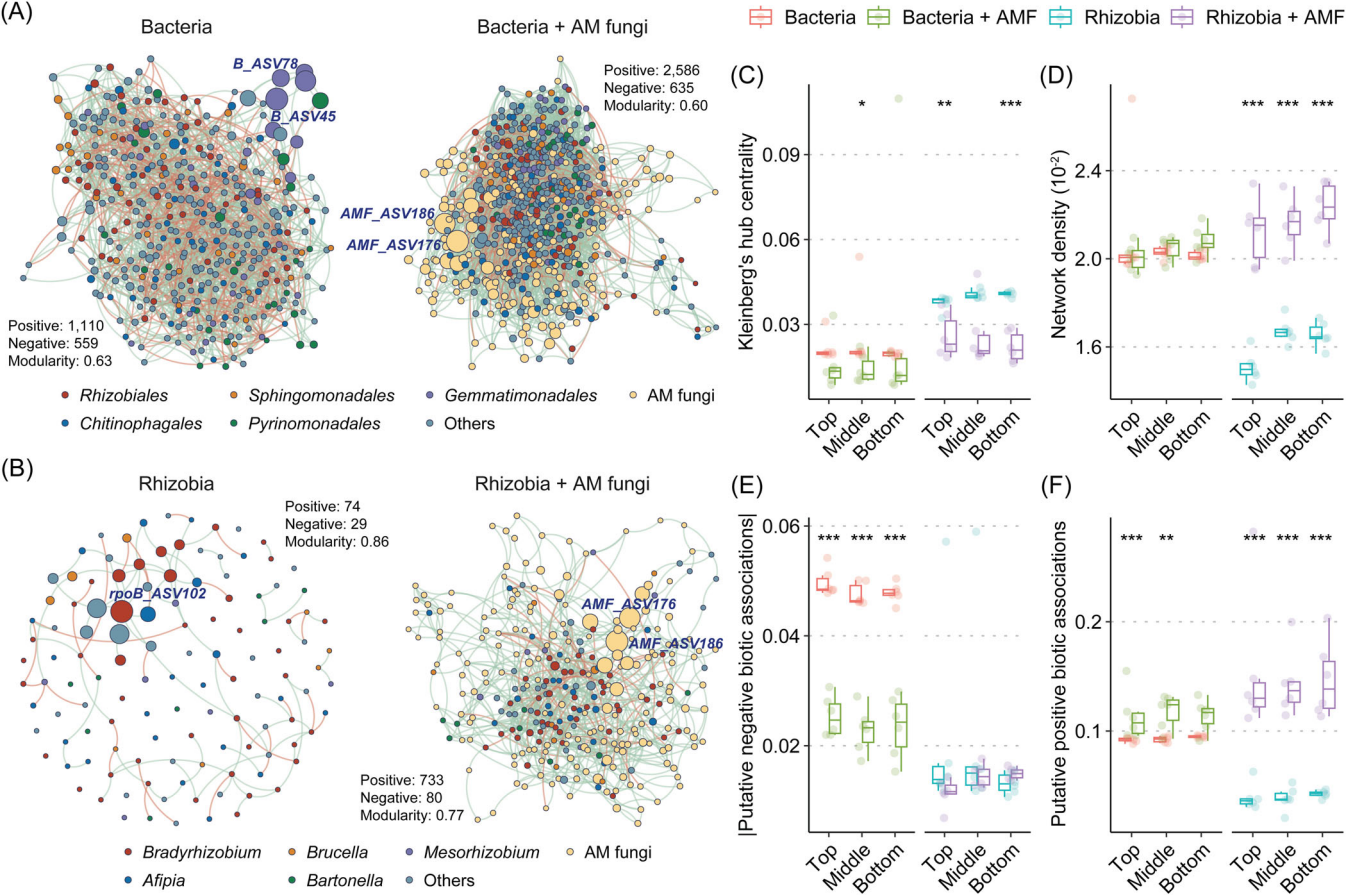
Influence of arbuscular mycorrhizal (AM) fungal interactions on ecological networks of bacteria and rhizobia. Differences in single‐ and cross‐kingdom (with the addition of AM fungi) networks of bacteria (A) and rhizobia (B). Nodes are shown in different colors at the order and genus levels for bacteria and rhizobia, respectively, with light yellow nodes indicating AM fungal amplicon sequence variants (ASVs). Node size represents Kleinberg's hub centrality of individual ASVs, whose score ≥0.7 were identified as keystone taxa (dark blue text). Detailed topological characteristics of networks are listed in Table 1. Comparisons of network centrality (C), density (D), putative negative (E), and positive biotic associations (F) at different slope positions (*n* = 6). Asterisks denote the significant differences between single‐ and cross‐kingdom networks of bacteria or rhizobia. *p < 0.05; **p < 0.01; ***p < 0.001.

To explore the role of biological interactions in the coexistence of root‐associated microbiota, we constructed both single‐ and cross‐kingdom networks for bacteria and rhizobia with and without AM fungi. The results suggested that the networks became more interconnected and showed reduced modularity when AM fungal interactions were incorporated (Figure 3A,B). Notably, in cross‐kingdom networks, topological metrics such as edge numbers, average degrees, and clustering coefficients were consistently higher compared to single‐kingdom networks (Table 1). Of these newly added edges, bacteria–AM fungi (24.2%) and rhizobia–AM fungi (28.7%) constituted substantial proportions in their respective cross‐kingdom networks. The within‐kingdom edges also increased following the addition of AM fungal interactions, with those in bacterial networks and rhizobial networks rising from 1669 to 2095 and from 103 to 269, respectively (Table 1).

**Table 1:** Topological characteristics of total networks (including single‐ and cross‐kingdom) and subsets of cross‐kingdom networks.

Network type	Node number	Edge number	Average degree	Average path length	Network diameter	Network density	Clustering coefficient
Total networks							
Bacteria	415	1669	8.04	0.065	0.447	0.019	0.077
Rhizobia	121	103	1.70	0.329	0.892	0.014	0.060
Bacteria + AM fungi	597	3221	10.8	0.047	0.521	0.018	0.082
Rhizobia + AM fungi	303	813	5.37	0.176	1.195	0.018	0.119
Subsets of cross‐kingdom networks							
Bacteria–bacteria^a^	415	2095	10.1	0.046	0.445	0.024	0.083
AM fungi–AM fungi^a^	182	348	3.89	0.404	2.439	0.022	0.190
Bacteria–AM fungi^a^	597	778	3.12	0.182	1.140	0.006	0
Rhizobia–rhizobia^b^	121	269	4.56	0.149	0.960	0.039	0.129
AM fungi–AM fungi^b^	182	311	3.44	0.500	2.276	0.019	0.179
Rhizobia–AM fungi^b^	303	233	2.08	0.501	1.871	0.009	0

Abbreviation: AM, arbuscular mycorrhizal.

^a^ Subsets of the bacteria + AM fungi network.

^b^ Subsets of the rhizobia + AM fungi network.

In general, the incorporation of AM fungal interactions significantly enhanced the density of rhizobial network but led to a reduction in Kleinberg's hub centrality of amplicon sequence variants (ASVs), irrespective of slope position (Figure 3C,D). This resulted in a notable shift in the identified keystone taxa, from *Bradyrhizobium* ASV102 to AM fungal ASV176 and ASV186 (Figure 3B). Simultaneously, these two ASVs from AM fungi were also recognized as keystone species in the cross‐kingdom network for bacteria (Figure 3A), assuming the topological roles of network connectors (Figure S3). More importantly, based on the quantification of community‐level putative biotic associations, the addition of AM fungal interactions significantly reduced negative biotic associations in bacterial networks and increased positive associations in both bacterial and rhizobial networks (*p* < 0.001; Figure 3E,F). The strength of positive associations was much higher than that of negative associations, aligning with the ratio of positive and negative edges (Figure 3A,B).

### Microbial community assembly and its drivers across the slope

**Figure 4:**
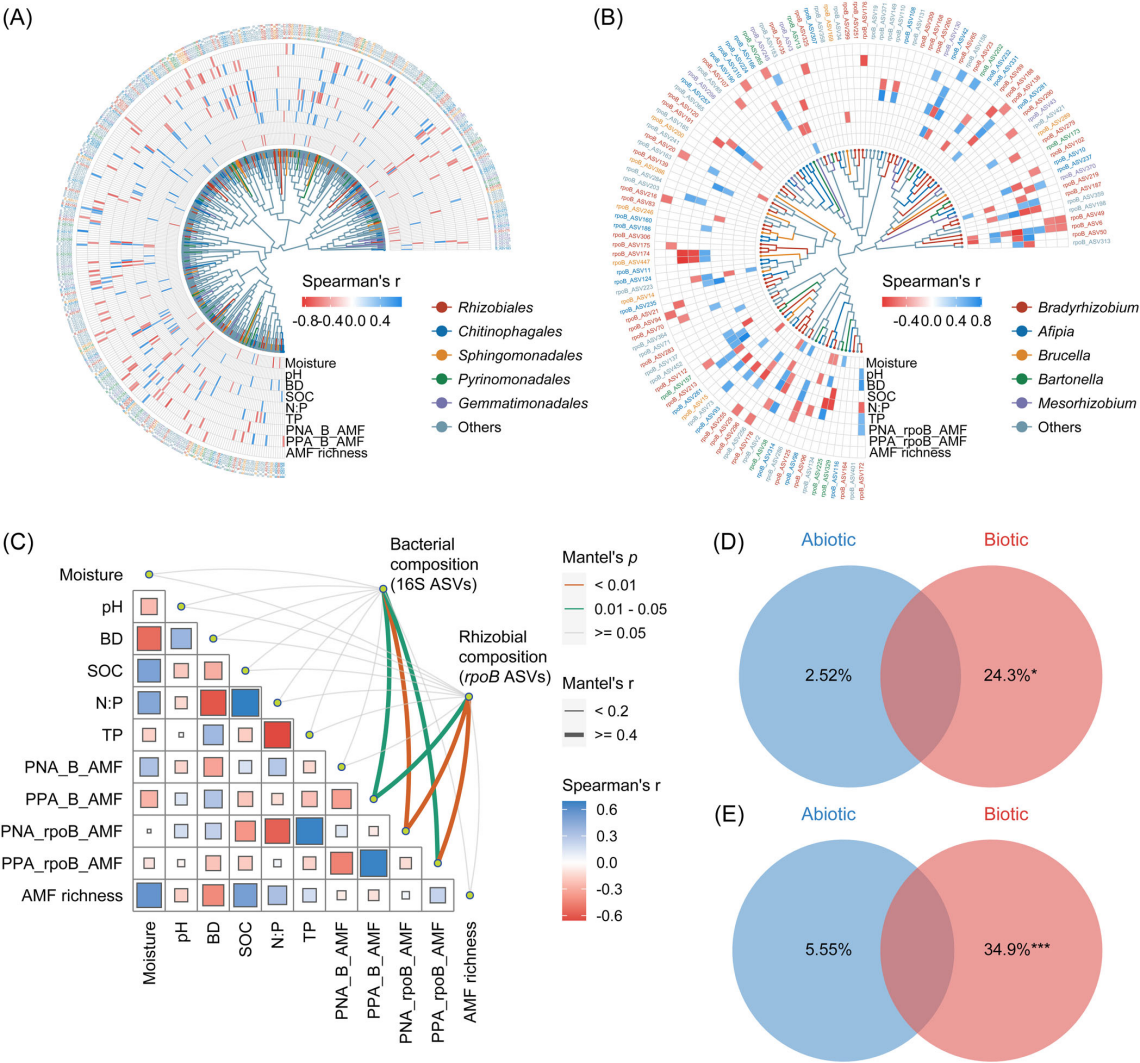
Phylogeny and drivers of dominant bacterial and rhizobial compositions on the slope. Heatmap and phylogenetic tree of dominant amplicon sequence variants (ASVs) for bacteria (A) and rhizobia (B) at the order and genus levels, respectively. The color scale indicates significant Spearman's correlations (*p* < 0.05) between ASVs and drivers (abiotic or biotic factors). (C) Relationships of bacterial and rhizobial compositions (Bray–Curtis distance) with abiotic and biotic factors. Pairwise correlations of these drivers are shown using Spearman's correlation coefficients. Edge width and color denote the Mantel's correlation coefficient and statistical significance, respectively. Proportions of abiotic and biotic factors explaining the variation in the community composition of bacteria (D) and rhizobia (E). *p < 0.05; ***p < 0.001. AMF richness, arbuscular mycorrhizal fungal richness; BD, bulk density; N:P, soil nitrogen‐to‐phosphorus ratio; PNA_B_AMF and PPA_B_AMF, putative negative and positive associations between bacteria and AM fungi, respectively; PNA_rpoB_AMF and PPA_rpoB_AMF, putative negative and positive associations between rhizobia and AM fungi, respectively; SOC, soil organic carbon; TP, total phosphorus.

We delved deeper into the impact of AM fungal interactions on microbial community assembly and composition across the slope. The phylogenetic tree and correlation analysis showed that AM fungal richness and cross‐kingdom biotic associations significantly influenced the majority of dominant taxa, with *Bradyrhizobium* being the main responsive taxon (Figure 4A,B). Mantel's test demonstrated a robust linkage between biotic associations and the composition of microbial communities, particularly the negative and positive associations between rhizobia and AM fungi (*p* < 0.01; Figure 4C). In addition, several abiotic factors showed significant correlations with microbial abundance and associations, accounting for a certain proportion of the variation in microbial compositions (2.52%–5.55%; Figure 4). Specifically, soil N:P was directly proportional to AM fungal richness but inversely proportional to negative rhizobia–AM fungi associations; soil moisture was largely associated with high AM fungal richness (Figure 4C). However, in general, biotic factors contributed much more to the explained variations in bacterial and rhizobial compositions by 24.3% and 34.9%, respectively (Figure 4D,E).

Null model analysis suggested that dispersal limitation contributed the largest proportion to the community assembly of bacteria (88.9%) and rhizobia (61.4%) across the slope, followed by drift (5.23%–34.0%; Figure S4). The varying AM fungal interactions were confirmed to significantly influence the ratio of these stochastic processes, manifesting as a shift from bacterial and rhizobial stochasticity to variable and homogeneous selections, respectively (Figure S5).

### Linking root‐associated microbial associations to slope multifunctionality

**Figure 5:**
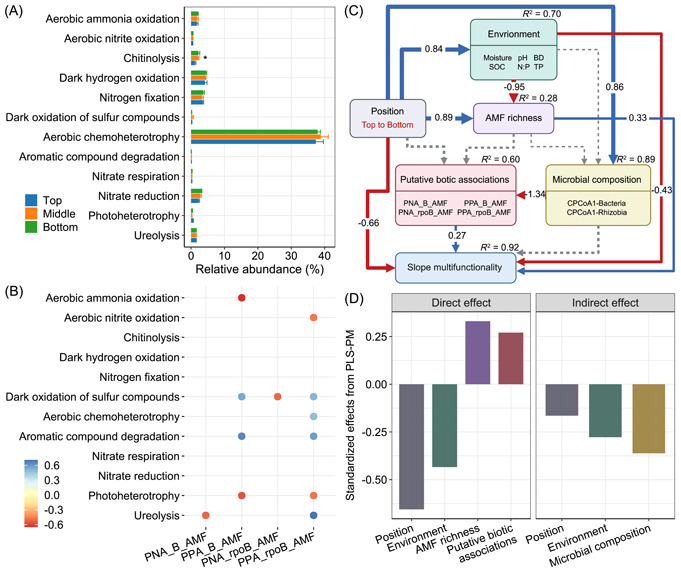
Linkages between microbial associations and multifunctionality across the slope. (A) Metabolic functions of the whole bacterial kingdom. Different colors represent different slope positions (top, middle, and bottom). Values are the means ± standard error (*n* = 6). *p < 0.05. (B) Significant correlations (Spearman's *p* < 0.05) between bacterial metabolic functions and cross‐kingdom biotic associations. (C) Partial least‐squares path modeling (PLS‐PM) identifying pathways whereby microbial properties and other factors affect slope multifunctionality. Red and blue arrows indicate negative and positive effects, respectively, with gray dotted lines representing nonsignificant effects (*p* > 0.05). Numbers inside the arrows indicate standardized effect sizes. *R* ^2^ denotes the proportion of variance explained. (D) Standardized direct and indirect effects from PLS‐PM. Only the standardized effects of factors with significant pathways on slope multifunctionality are shown. AMF richness, arbuscular mycorrhizal fungal richness; BD, bulk density; CPCoA 1, the first axis of constrained principal coordinate analysis; N:P, soil nitrogen‐to‐phosphorus ratio; PNA_B_AMF and PPA_B_AMF, putative negative and positive associations between bacteria and AM fungi, respectively; PNA_rpoB_AMF and PPA_rpoB_AMF, putative negative and positive associations between rhizobia and AM fungi, respectively; SOC, soil organic carbon; TP, total phosphorus.

Across the slope, 12 bacterial metabolic functions were detected, involving nutrition and energy metabolisms (Figure 5A). Aerobic chemoheterotrophy emerged as the predominant metabolic pathway, constituting approximately 40% of the total functional abundance, followed by dark hydroxide oxidation and N fixation. Despite their low relative abundance, certain metabolic functions were closely linked to cross‐kingdom biotic associations (Figure 5B). For example, the relative abundances of dark oxidation of sulfur compounds and aromatic compound degradation increased with the strength of positive associations, while aerobic ammonia/nitrite oxidation and photoheterotrophy decreased (*p* < 0.05). We also explored the role of biotic associations in driving slope multifunctionality. The results showed that the stronger positive associations between rhizobia and AM fungi supported the activity of alkaline phosphatase (AP); in contrast, their negative associations inhibited DOC, MBC, and NAG, thus leading to a reduction in multifunctionality (Figure S6).

Through the construction of partial least‐squares path modeling (PLS‐PM) that incorporated both microbial properties and abiotic factors, we determined that slope multifunctionality was directly supported by AM fungal richness and biotic associations, with indirect regulation by microbial composition (Figure 5C,D). Additionally, slope position and environmental variables jointly influenced multifunctionality by interacting with microbial properties, particularly AM fungal richness and rhizobial composition (Figure 5C,D and Table S3). Collectively, all predictors accounted for a considerable variance (91.9%) in slope multifunctionality according to our generalized linear mixed model (GLMM).

## DISCUSSION

The influence of slope position on biogeochemical cycling within degraded ecosystems is a complex process shaped by soil erosion dynamics [15]. In this study, we elucidated this complexity by unraveling a nuanced pattern of multifunctionality across the slope, with its multiple facets decreasing toward lower slope positions (Figure 1). This pattern, intriguingly, aligned with the reduction in soil N:P from the top to the bottom position (Figure 2G and Table S2), challenging the general understanding that aggravated P limitation hampers ecosystem functions. We attribute this to stoichiometric‐driven alterations in microbial diversity and composition [33]. Stochiometric imbalances generally trigger adaptive mechanisms in microbes, leading to adjustments in C use efficiency and enzymatic production [34]. Despite reduced plant production at upper slope positions with high N:P imbalance, these microbial adaptations profoundly improved functions related to microbially driven C pools and organic matter decomposition (Figure 1A,C and Tables S1 and S2). Additionally, internal nutrient cycling may be inherent in P‐limited forest ecosystems [35], which is characterized by increased plant investment into root‐associated microbiota for the extra mineralization of available nutrients [36]. Within the root‐associated microbiota, AM fungi that provide most of the P needs for plants tended to be enriched and pivotal in coping with high N:P imbalance (Figures 2C and 4C).

Note that the influence of soil moisture changes on slope multifunctionality, while not statistically significant in random forest analysis, cannot be disregarded (Figure 2G and Tables S2 and S3). Soil moisture has been consistently recognized as a critical factor influencing microbial community and functionality, particularly within the context of deep‐rooted species like *R. pseudoacacia* in degraded landscapes [13, 37]. Our previous findings also highlighted that *R. pseudoacacia* positioned lower on the slope showed higher growth advantages and rhizosphere effects [16], potentially resulting in enhanced soil moisture depletion [38]. This process mirrored the gradient of declining soil moisture from the top to the bottom of the slope (Table S2). In contrast, the enrichment of AM fungi at upper slope positions provided *R. pseudoacacia* with better water retention capacity [6], as evidenced by the strong positive correlation between soil moisture and AM fungal richness (Figure 4C). Such findings imply a strategic investment of plants in establishing biotic relationships, particularly in the face of abiotic challenges like gravity‐driven resource transportation along the slope. Consequently, the intricate plant–microbe interactions, modulated by the availability of key resources such as water and nutrients, play a decisive role in the multifunctionality of the eroded slope.

Symbiotic and heterotrophic microbes are crucial yet less known contributors to the performance of *R. pseudoacacia* in rehabilitating degraded ecosystems [1, 6]. Our investigation of the root‐associated microbiota suggested that the richness of AM fungi and the composition of bacteria and rhizobia significantly drove slope multifunctionality (Figure 2 and Figure S2), consistent with the established roles of mycorrhizal associations and dominant bacterial taxa [39, 40]. Notably, separate amplicon sequencing identified Rhizobiales and *Bradyrhizobium* as the dominant bacterial order and rhizobial genera, respectively (Figure S1). Based on the relative abundance of 16S *rRNA* gene sequences, our quantification of the absolute *rpoB* gene abundance revealed a marked amplification and selection of rhizobia within the *R. pseudoacacia* rhizosphere [29], especially at the top of the slope (Figure 2B and Figure S1A). This reverified the enhanced colonization of AM fungi at relevant locations (Figure 2C), and simultaneously supported our first hypothesis, as AM symbioses fulfill the great demand of P by plants with N‐fixing symbionts [41]. Regarding bacteria dominated by heterotrophic clades, they thrived at lower slope positions with the increased availability of plant‐ and litter‐derived organic matter as food sources (Figure 2A).

In turn, AM fungi were proven to mediate the balance of microbial community assembly and stabilize cross‐kingdom networks. We revealed that dispersal limitation predominantly shaped the assembly of both bacterial and rhizobial communities (Figure S4), probably due to their constrained growth or spore production by limited P and moisture availability. Under the circumstances, the presence of AM fungi significantly enhanced network connectivity and within‐ and cross‐kingdom links, reinforcing positive biotic associations (Table 1 and Figure 3). This resonates with a broad range of evidence that more pronounced microbial co‐occurrence patterns are highly linked to dispersal limitation in water‐ and nutrient‐deficit habitats [42, 43]. As such, AM fungal richness and biotic associations strengthened the homogeneous sorting effects on rhizobia and stabilized the whole bacterial kingdom (Figure S5). Furthermore, AM fungal interactions contributed a much larger proportion than abiotic factors to the phylogeny and composition of bacteria and rhizobia (Figure 4), which confirmed the indispensable role of AM fungi in bacterial community assembly as we hypothesized [10, 42]. The significantly differed network complexity between single‐ and cross‐kingdom networks instead of among slope positions with obvious environmental heterogeneity also reaffirmed this conclusion (Figure 3C–F and Table S2).

Gemmatimonadales (bacteria) and *Bradyrhizobium* (rhizobia) were the keystone taxa in single‐kingdom networks (Figure 3A,B), demonstrating strong support for both ecosystem N accumulation and the ability to withstand low water availability [44, 45]. However, P limitation associated with N:P imbalance poses a threat to ecosystem restoration and sustainability [46, 47]. Notably, two AM fungal ASVs assumed the role of connectors and keystone species in cross‐kingdom networks (Figure 3A,B and Figure S3), highlighting the pivotal role of AM fungi in supporting essential ecosystem functions [48]. The transition in keystone taxa and microbial co‐occurrence patterns underlines how *R. pseudoacacia* enriches functionally beneficial microbiota, spontaneously forming an AM fungal‐centered network to enhance nutrient acquisition and alleviate P limitation [49, 50]. Enhanced P uptake capacity by AM fungi then initiates a cascade of shifts, including increased N fixation and thus symbioses with rhizobia, which has a positive feedback effect on establishing AM mutualisms. Such co‐evolution of AM fungi and rhizobia within the root holobiont of *R. pseudoacacia* suggests a tripartite partnership (i.e., legume–AM fungi–rhizobia symbiotic association [6]), potentially making significant contributions to slope multifunctionality under P limitation.

The tripartite symbiotic association has been demonstrated to enhance legume fitness through mycorrhizal promotion of rhizobial nodules [6, 51]. This study revealed that less competition between rhizobia and AM fungi significantly increased slope multifunctionality, particularly in microbially driven C pools and chitin degradation (Figure S6A–E). More importantly, stronger rhizobia–AM fungi facilitations would unleash greater P mineralization potential (Figure S6F). Across the eroded slope with limited resources, such synergistic facilitations can also favor recalcitrant compound decomposition and optimize microbial metabolism by allocating substrates for chemoheterotrophs (Figure 5B). Moreover, the metabolic interdependence between these symbionts [52] further confirmed the selective enhancement of rhizobia and the significant spatial variation in microbial composition (Figure 2D,E and S5E,F). In the co‐evolution of AM fungi and rhizobia, their respective master regulators (PHR2 and NIN) act as the counterparts in a common signaling pathway, controlling the homeostasis of P versus nitrate [53]. Mechanistically, *R. pseudoacacia* may recruit more PHR2 to orchestrate its self‐development and cope with the N:P imbalance across the slope. While we have underscored the role of tripartite symbiosis in enhancing slope multifunctionality, it is important to acknowledge its context dependency, given the lack of studies on eroded slopes or similar landscapes. Future research should validate these findings across diverse ecological settings to better understand their implications for ecosystem functioning in degraded landscapes.

**Figure 6:**
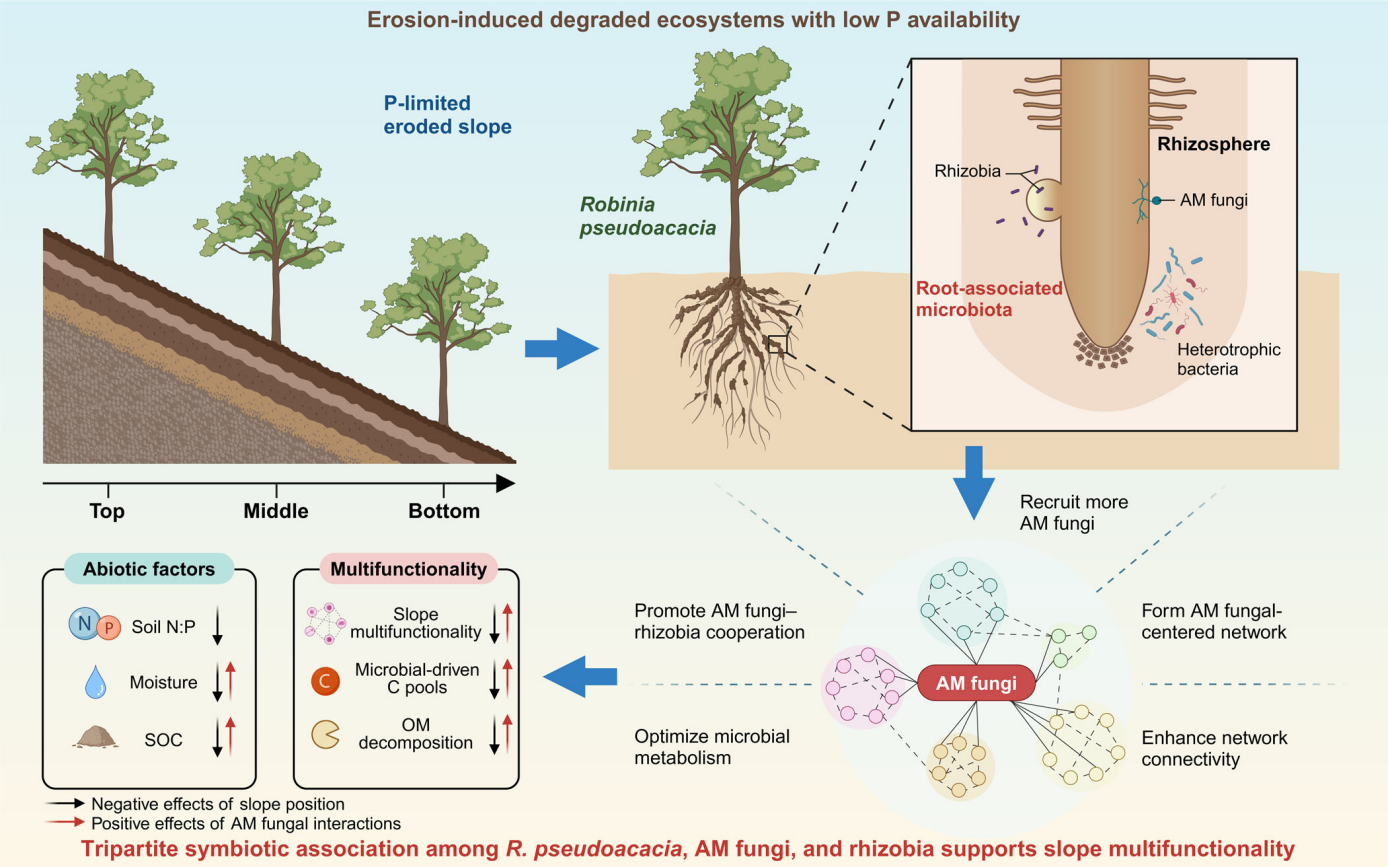
Conceptual diagram characterizing the indispensable role of arbuscular mycorrhizal (AM) fungal‐centered *Robinia pseudoacacia* root‐associated microbiota in restoring erosion‐prone ecosystems with low P availability. The diagram visually shows how *R. pseudoacacia* strategically recruits AM fungi and forms an AM fungal‐centered network in the rhizosphere to cope with P limitation on the slope. Black and red arrows indicate the negative effects of slope position and positive effects of AM fungal interactions on abiotic factors or multifunctionality of the eroded slope. N:P, soil nitrogen‐to‐phosphorus ratio; OM decomposition, organic matter decomposition; SOC, soil organic carbon.

Even after accounting for several abiotic factors, PLS‐PM and GLMM analyses robustly affirmed the support of AM fungal interactions for slope multifunctionality (Figure 5C,D and Table S3). As stated in our third hypothesis, regardless of resource distribution along the slope, the detrimental effects of P limitation on multifunctionality were counteracted by the direct contribution of AM fungal richness and biotic associations, notably the mutualism with rhizobia (Figure 6). This was corroborated by a recent study on secondary vegetation succession [54] and our result that a higher N:P imbalance correlated with increased AM fungi presence and less negative rhizobia–AM fungi associations (Figure 4C). Furthermore, the composition of bacteria and rhizobia accounted for a considerable proportion of slope multifunctionality, mediated through biotic associations (Figure 5C,D and Table S3). Echoing the Community Assembly and the Functioning of Ecosystems perspective [55], microbial community assembly and composition driven by AM fungal interactions emerge as key factors driving slope multifunctionality. Taken together, considering the profound impact of soil erosion on ecosystem services [26], our study emphasizes the irreplaceability and efficacy of AM fungal‐centered legume microbiota in the sustainable restoration of erosion‐prone ecosystems.

## CONCLUSION

This study provides empirical evidence that AM fungal interactions are strongly linked to the delivery of slope multifunctionality within *R. pseudoacacia* plantations, particularly in microbially driven C pools and organic matter decomposition. Across the eroded slope, AM fungal richness and the composition of bacteria and rhizobia jointly drive spatially explicit multifunctionality, with AM fungal interactions playing a pivotal role in the assembly and composition of bacterial and rhizobial communities. In response to resource limitations, especially P and moisture, *R. pseudoacacia* strategically recruits AM fungi, forming an AM fungal‐centered network in the rhizosphere. More importantly, the *R. pseudoacacia*–AM fungi–rhizobia tripartite symbiotic association robustly supports slope multifunctionality, even after accounting for abiotic factors (Figure 6). We must highlight that this association is derived from an increased investment of *R. pseudoacacia* into biotic relationships and paramount to its successful use to restore degraded lands if the rainfall is sufficient to maintain the forest community. However, historically, a disproportionate number of legume woody species have been utilized in the forest restoration of degraded lands. Minimally, these findings illuminate vital clues for rehabilitating erosion‐induced degraded ecosystems and the indispensable biodiversity and services reliant on these ecosystems.

## METHODS

### Study site

The study site is located in the Gutun watershed on the central Loess Plateau, China (36°46′ N, 109°48′ E), falling within the warm temperate monsoon climate zone (Figure S7). The mean annual temperature and precipitation stand at 10.6°C and 531 mm, respectively, marked by an uneven rainfall pattern primarily occurring from July to September. Our experimental design closely follows the methodology outlined in our previous study [16]. Specifically, we focused on a slope of *R. pseudoacacia* plantation within the watershed, characterized by loessial soil and a gradient of 26°.

On this slope, we demarcated an area of 60 m × 30 m, and conducted surveys along three parallel transects, each transect with equal length (60 m) spaced at an interval of 10 m from left to right. Building on these 60 m transects, in each one, three positions (top, middle, and bottom) were established at an interval of 20 m and further divided into two plots (Figure S7). Consequently, there were a total of 18 plots (10 m × 10 m) spanning the entire slope, with each slope position boasting six replications.

### Plant and soil sampling

In September 2019, we implemented an S‐shaped approach to collect composite samples (from five cores) at each plot, encompassing both plant tissues and soil samples. The plant tissues comprised mature leaves and litter. For soil samples, we adopted a comprehensive strategy by collecting rhizosphere and bulk soils. This involved digging up the roots, sieving the soils through a 2 mm sieve, and segregating them following the standard procedure [56].

To elaborate, rhizosphere soils were meticulously brushed off from the soils adhering closely to the roots, and were stored at −80°C for subsequent DNA extraction. Bulk soils were dislodged directly from the roots, with each sample being split into two subsamples. One subsample was promptly stored at 4°C immediately for later assessments of ecosystem functioning variables, while the other subsample was subjected to natural air‐drying for analyses of physicochemical properties.

### Soil physicochemical analyses

We assessed abiotic factors closely tied to the activities of root‐associated microbiota, encompassing soil moisture, bulk density (BD), pH, SOC, total nitrogen (TN), and total phosphorus (TP). Soil BD was analyzed using a bulk sampler and a 5‐cm‐diameter stainless‐steel cutting ring [37]. The determination of other abiotic factors followed previously established methods [16].

Briefly, soil moisture content was determined by oven‐drying 10 g of fresh soil at 105°C. Soil pH was determined with a pH meter (InsMark IS126) in a 1:2.5 mass‐to‐volume slurry. SOC was determined using the sulfuric acid–potassium dichromate external heating method. TN and TP were determined using standardized protocols, namely, the Kjeldahl technique and the molybdenum antimony colorimetric method, respectively. Due to the observed multicollinearity between SOC and TN (Spearman's coefficient = 0.83), we calculated soil N:P as the ratio of TN to TP and substituted it for TN in statistical analyses.

### Ecosystem functioning measures

We quantified 12 ecosystem functions associated with four essential services, following the approach of previous studies [39, 57]. These functions comprised microbially driven C pools (DOC and MBC), nutrient cycling (soil total dissolved N (TDN), available phosphorus (Olsen‐P), microbial biomass N (MBN), and MBP), organic matter decomposition (BG, NAG, LAP, and AP), and plant production (leaf C and litter C contents).

Leaf and litter C contents were determined by oven‐drying at 60°C to a constant weight, followed by grinding and potassium dichromate external heating. The concentrations of DOC and TDN were extracted with potassium sulfate (K_2_SO_4_) and determined using a total C analyzer (Elementar). Olsen‐P was extracted with sodium bicarbonate (NaHCO_3_) and determined using a spectrophotometer (Hitachi UV2300). After fumigating 45 g of fresh soil with chloroform for 24 h, MBC and MBN were extracted with K_2_SO_4_ and determined using the total C analyzer, while MBP was extracted with NaHCO_3_ and determined using the spectrophotometer [58, 59]. Conversion factors of 0.45, 0.54, and 0.40 were applied for calculating MBC, MBN, and MBP, respectively [60]. The activities of BG, NAG, LAP, and AP were determined from 1 g of fresh soil using a fluorometric method, with 7‐amino‐4‐methylcoumarin and methylumbelliferone as labeled substrates for LAP and other enzymes, respectively [61].

To facilitate analysis, we standardized individual 12 ecosystem functions (Table S1) by *z*‐transformation to yield the multifunctionality index for each plot across the slope [62, 63]. This index encapsulates the capability to simultaneously deliver multiple ecosystem services [31]. Three facets of multifunctionality were considered: (i) multiple single functions; (ii) average multifunctionality, calculated by averaging the 12 standardized ecosystem functions; and (iii) multidimensional functioning, assessed by three principal axes of PCA that can handle the potential collinearity among measured ecosystem functions [31, 62].

### Amplicon sequencing and molecular analyses

Genomic DNA extraction from each of the 18 rhizosphere soil samples was conducted using the ALFA‐Soil DNA Extraction Mini Kit following the manufacturer's instructions. To elucidate the richness and community composition of root‐associated microbiota, we amplified the bacterial 16S *rRNA*, rhizobial *rpoB*, and *Glomeromycota* nuclear 18S *rRNA* genes using specific primer pairs: 341F (5′‐CCT ACG GGA GGC AGC AG‐3′)/806 R (5′‐GGA CTA CHV GGG TWT CTA AT‐3′), rpoB1479F (5ʹ‐GAT CGA AAC GCC GGA AGG‐3ʹ)/rpoB1831R (5ʹ‐TGC ATG TTC GAG CCC AT‐3ʹ) [29], and NS31 (5ʹ‐TTG GAG GGC AAG TCT GGT GCC‐3ʹ)/AMDGR (5ʹ‐CCC AAC TAT CCC TAT TAA TCA T‐3ʹ) [64]. Amplicon sequencing and bioinformatic processing were performed using the Illumina MiSeq platform and DADA2 in the QIIME2 pipeline, respectively [65]. Phylotypes were identified at a 100% similarity level, denoted as ASVs. Following demultiplexing and quality filtering, a total of 50,154 bacterial ASVs, 4542 rhizobial ASVs, and 2807 AM fungal ASVs were finally retained and clustered.

Bacterial ASVs were categorized using the SILVA SSU132 database [66]. For rhizobial ASVs, only the *rpoB* gene sequences from the *Proteobacteria* clade were retained and annotated at the genus level [29]. In the case of AM fungi, *Glomeromycota* 18S *rRNA* gene sequences were assigned by performing BLAST against the MaarjAM database [67]. The prediction of bacterial metabolic functions was accomplished by assigning 16S *rRNA* amplicon sequences to the FAPROTAX database [68].

### Ecological network and biotic association inference

The SPIEC‐EASI algorithm, which was robust in handling the bias of community compositionality [69], was used to infer both single‐ and cross‐kingdom networks of root‐associated microbiota (AM fungi, rhizobia, and bacteria). For each microbiota, dominant ASVs occurring in more than 25% of all samples with a relative abundance exceeding 0.001% were retained for network analyses [70]. Using the “microeco” R package version 0.17.0 [71], these ASV tables were either used separately or merged to construct single‐ or cross‐kingdom networks. Topological characteristics of both network types, encompassing the number of nodes and edges (positive and negative), average degree, average path length, modularity, network diameter, and density, were computed using the “igraph” R package version 1.4.0 [72]. Using the “subgraph” function, we further calculated the required topological characteristics of individual sample subnetworks or subsets of cross‐kingdom networks. To classify topological roles of nodes in cross‐kingdom networks, their within‐module connectivity (*Z*) and among‐module connectivity (*P*) were calculated and used according to widely adopted criteria [63, 73]. Specifically, module hubs (*Z* > 2.5, *P* < 0.62), network hubs (*Z* > 2.5, *P* > 0.62), and connectors (*Z* < 2.5, *P* > 0.62) were categorized, and other nodes were identified as peripherals. Keystone taxa were pinpointed utilizing Kleinberg's hub centrality score with a threshold of 0.7 [74]. Community‐level biotic associations were quantified by multiplying matrices of ASVs and their positive and negative connectedness using the “qcmi” R package version 0.1.2 [70].

### Null model analysis for assembly processes

A null modeling‐based approach, QPEN (Quantifying assembly Processes based on Entire‐community Null model analysis), was used to estimate the assembly processes of bacterial and rhizobial communities [75, 76]. This approach infers selection from phylogenetic metrics using specific thresholds, such as the *β*‐nearest taxon index (*β*NTI) and Bray–Curtis‐based Raup–Crick (RC_Bray_). A dominance of deterministic processes (variable or homogeneous selection) was indicated when |*β*NTI| > 2, whereas stochastic processes governed the community assembly when |*β*NTI| < 2. In this case, |RC_Bray_| > 0.95 and |RC_Bray_| < 0.95 indicated the dominance of homogeneous dispersal or dispersal limitation and shift, respectively [43]. Calculations of *β*NTI and RC_Bray_ were performed with 1000 randomizations using the “iCAMP” R package version 1.5.12 [75].

### Statistical analyses

Linear mixed‐effects models, with the plot as a random effect, were used to analyze the fixed effects of slope position on the richness of root‐associated microbiota and ecosystem functions (individual functions, multiple dimensions of functions, and multifunctionality) using the “lmerTest” R package version 3.1‐3 [77]. PCA was conducted on the spatial variation of multiple ecosystem functions across the slope. From the PCA results, explained variances and eigenvalues of first three axes were extracted to evaluate multidimensional functioning. PERMANOVA with 999 permutations was conducted to test significant differences in PCA eigenvalues among slope positions, using the “vegan” R package version 2.6‐4 [78]. CPCoA based on Bray–Curtis dissimilarities was performed to explore the maximum explained variation in the composition of each microbiota by slope position. Using the “rfPermute” R package version 2.5.1 [79], we used a random forest analysis comprising 500 trees and 1000 permutations to identify the major factors driving slope multifunctionality. The significances of these factors and the random forest model were assessed based on percentage increase in the mean square error and using the “A3” R package version 1.0.0 [80], respectively. Spearman's correlation analyses were used to test relationships among microbiota, environmental factors, and ecosystem functioning across the slope. Mantel's tests were performed to determine the influence of individual drivers (i.e., abiotic and biotic factors) on the community assembly and composition of bacteria and rhizobia. Variation partitioning analysis was carried out to compare the relative contribution of abiotic and biotic factors to bacterial and rhizobial compositions separately. PLS‐PM was used to ascertain the pathways whereby factors directly or indirectly affected slope multifunctionality, using the “plspm” R package version 0.5.0 [81]. The reliability and validity of PLS‐PM were evaluated and accepted by three standards: goodness of fit = 0.6, average variance extracted > 0.5, and Cronbach's *α* > 0.7 [82]. With the plot as a random effect, a GLMM was used to additionally estimate the fixed effects and independently explained variations of factors in the “glmm.hp” R package version 0.1‐0 [83]. All statistical analyses and visualizations were performed using R statistical software version 4.1.2 [84].

## AUTHOR CONTRIBUTIONS

Linchuan Fang and Tianyi Qiu designed the study. Tianyi Qiu performed the analyses and wrote the manuscript. Josep Peñuelas, Yinglong Chen, and Jordi Sardans discussed the design, the analyses, and the results, providing conceptual inputs to improve the study and the manuscript. Jialuo Yu performed the experiments and collected the data. Zhiyuan Xu, Qingliang Cui, Ji Liu, Yongxing Cui, Shuling Zhao, Jing Chen, and Yunqiang Wang advised on the interpretation of the results and revised the manuscript. All authors have read the final manuscript and approved it for publication.

## CONFLICT OF INTEREST STATEMENT

The authors declare no conflict of interest.

## Supporting information


**Figure S1:** Relative abundance of top 10 bacterial orders (A) and rhizobial genera (B) at three slope positions.
**Figure S2:** Heatmap showing the significant Spearman's correlations (*p* < 0.05) between microbial diversity and composition and ecosystem functions within multiple important services (microbially driven C pools, nutrient cycling, organic matter decomposition, plant production, and multifunctionality).
**Figure S3:** Topological roles of bacterial, rhizobial, and AM fungal nodes in the cross‐kingdom networks.
**Figure S4:** Relative contribution of different assembly processes in shaping bacterial and rhizobial communities across the slope.
**Figure S5:** Responses of bacterial and rhizobial *β*‐nearest taxon index (*β*NTI) to changes in AM fungal richness and cross‐kingdom biotic associations tested by Mantel's tests.
**Figure S6:** Putative biotic associations between rhizobia and AM fungi driving slope multifunctionality.
**Figure S7:** Experimental design diagram on an eroded slope of a *Robinia pseudoacacia* plantation.

**Table S1:** Distribution of ecosystem functions related to four important services on the slope using linear mixed‐effects models.
**Table S2:** Distribution of environmental variables on the slope using linear mixed‐effects models.
**Table S3:** Hierarchical partitioning analysis clarifying the contribution of individual predictors to slope multifunctionality in the generalized linear mixed‐effects model (GLMM).

## Data Availability

All data generated and analyzed in this study are available in the NCBI Sequence Read Archive under the BioProject PRJNA1083417 (http://www.ncbi.nlm.nih.gov/bioproject/1083417) or included in Supporting Information. The data and scripts have been deposited in GitHub (https://github.com/TianyiQiu13/Arbuscular-mycorrhizal-fungal-interactions). Supporting Information (figures, tables, scripts, graphical abstract, slides, videos, Chinese translated version, and update materials) may be found in the online DOI or iMeta Science http://www.imeta.science/.
