# Supplementary material for: Arbuscular mycorrhizal fungal interactions bridge the support of root‐associated microbiota for slope multifunctionality in an erosion‐prone ecosystem
Source: Imeta. 2024 Mar 25;3(3):e187. doi: 10.1002/imt2.187 (PMC11183171; doi:10.1002/imt2.187)
Supplement: Supplementary file 1 — Figure S1: Relative abundance of top 10 bacterial orders (A) and rhizobial genera (B) at three slope positions. Figure S2: Heatmap showing the significant Spearman's correlations (p < 0.05) between microbial diversity and composition and ecosystem functions within multiple important services (microbially driven C pools, nutrient cycling, organic matter decomposition, plant production, and multifunctionality). Figure S3: Topological roles of bacterial, rhizobial, and AM fungal nodes in the cross‐kingdom networks. Figure S4: Relative contribution of different assembly processes in shaping bacterial and rhizobial communities across the slope. Figure S5: Responses of bacterial and rhizobial β‐nearest taxon index (βNTI) to changes in AM fungal richness and cross‐kingdom biotic associations tested by Mantel's tests. Figure S6: Putative biotic associations between rhizobia and AM fungi driving slope multifunctionality. Figure S7: Experimental design diagram on an eroded slope of a Robinia pseudoacacia plantation. [file IMT2-3-e187-s002.docx]

**Supporting information to
Arbuscular mycorrhizal fungal interactions bridge the support of root-associated microbiota for slope multifunctionality in an erosion-prone ecosystem**

**Running title**: Arbuscular mycorrhizal-centered restoration

Tianyi Qiu ^1,2,3^, Josep Peñuelas ^4,5^, Yinglong Chen ^1,2,6^, Jordi Sardans ^4,5^, Jialuo Yu ^7^, Zhiyuan Xu ^1,2^, Qingliang Cui ^8^, Ji Liu ^9^, Yongxing Cui ^10^, Shuling Zhao ^8^, Jing Chen ^11^, Yunqiang Wang ^12^, Linchuan Fang ^1,3,8,12*^

^1^State Key Laboratory of Soil Erosion and Dryland Farming on the Loess Plateau, Northwest A&F University, Yangling 712100, China

^2^College of Natural Resources and Environment, Northwest A&F University, Yangling 712100, China

^3^Key Laboratory of Green Utilization of Critical Non-metallic Mineral Resources, Ministry of Education, Wuhan University of Technology, Wuhan 430070, China

^4^Consejo Superior de Investigaciones Científicas, Global Ecology Unit Centre de Recerca Ecològica i Aplicacions Forestals-Consejo Superior de Investigaciones Científicas-Universitat Autònoma de Barcelona, Bellaterra 08193, Spain

^5^Centre de Recerca Ecològica i Aplicacions Forestals, Cerdanyola del Vallès, Catalonia 08290, Spain

^6^The University of Western Australia Institute of Agriculture, and School of Agriculture and Environment, The University of Western Australia, Perth 6009, Australia

^7^Key Laboratory of Ecosystem Network Observation and Modelling, Institute of Geographic Sciences and Natural Resources Research, Chinese Academy of Sciences, Beijing 100101, China

^8^Institute of Soil and Water Conservation, Chinese Academy of Sciences and Ministry of Water Resources, Yangling 712100, China

^9^Hubei Province Key Laboratory for Geographical Process Analysis and Simulation, Central China Normal University, Wuhan 430079, China

^10^Institute of Biology, Freie Universität Berlin, Berlin 14195, Germany

^11^Department of Cardiology, Renmin Hospital of Wuhan University, Wuhan 430060, China

^12^Chinese Academy of Sciences Center for Excellence in Quaternary Science and Global Change, Chinese Academy of Sciences, Xi’an 710061, China

*Correspondence: [flinc629@hotmail.com](mailto:flinc629@hotmail.com) (Linchuan Fang)


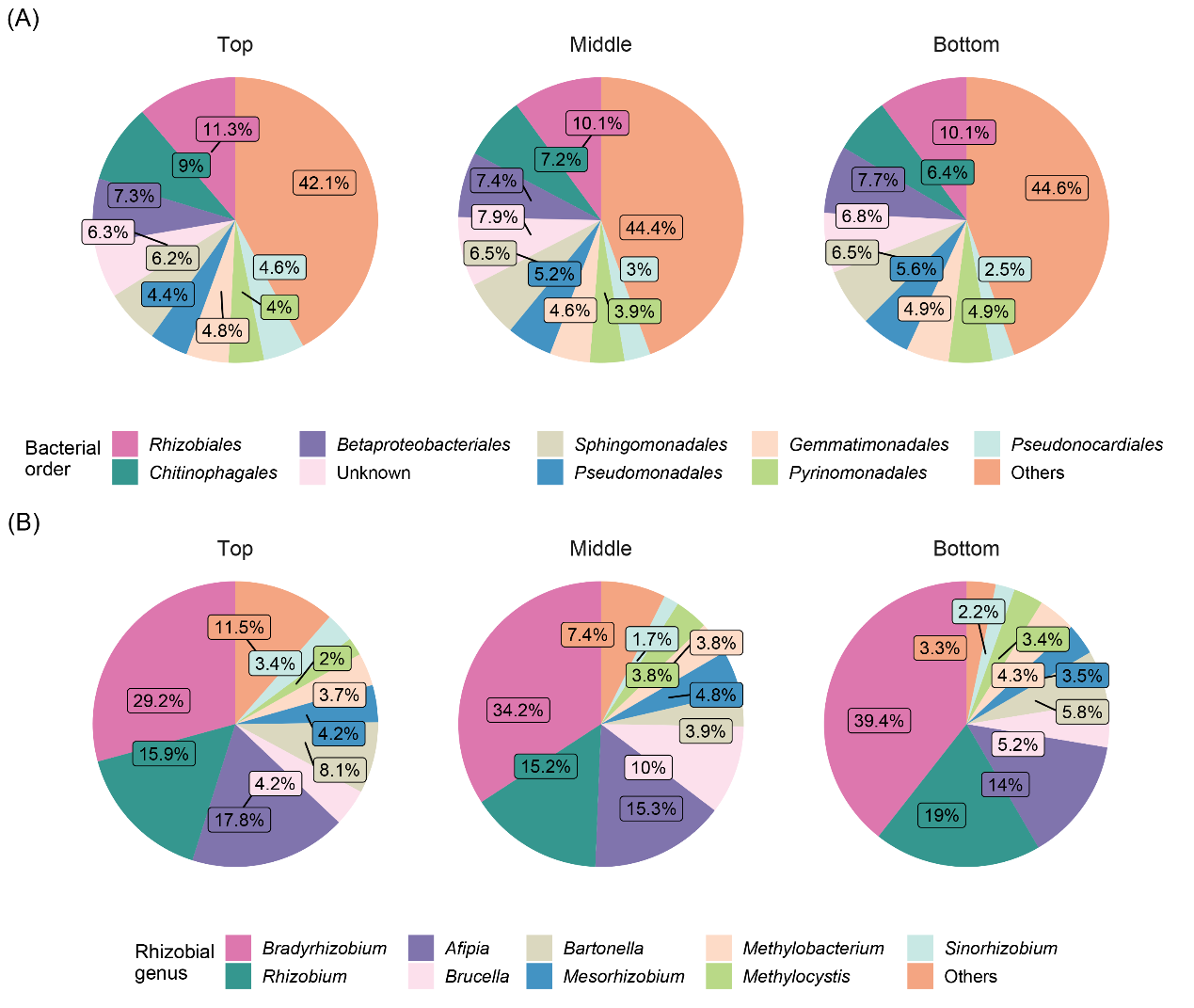


**Figure S1** Relative abundance of top 10 bacterial orders (A) and rhizobial genera (B) at three slope positions.


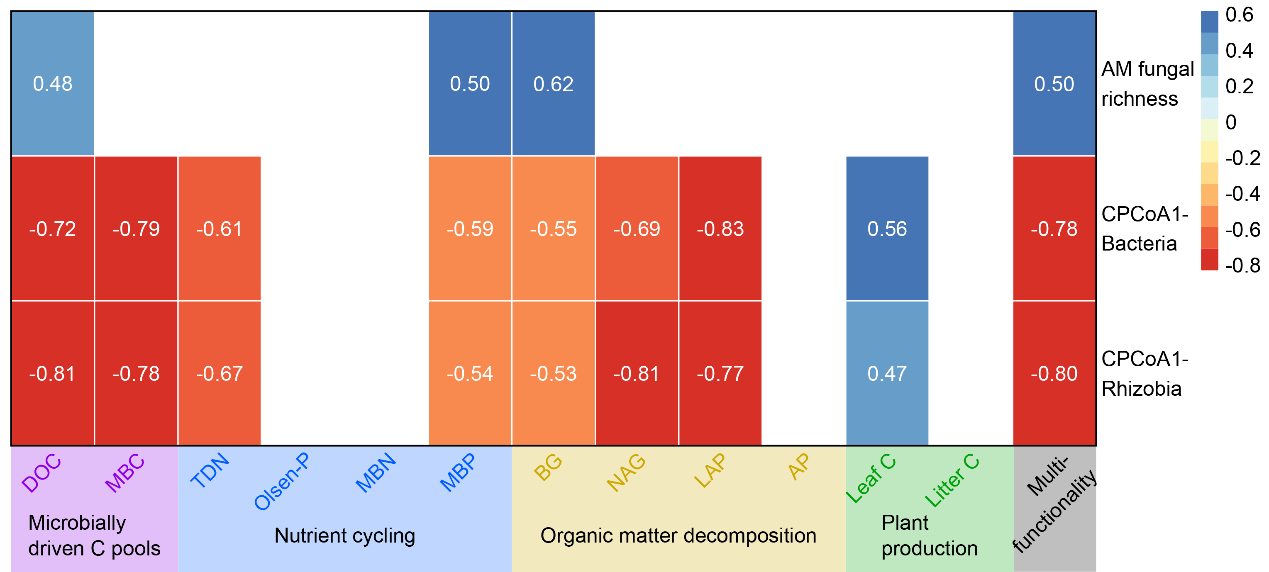


**Figure S2** Heatmap showing the significant Spearman’s correlations (*p* < 0.05) between microbial diversity and composition and ecosystem functions within multiple important services (microbially driven C pools, nutrient cycling, organic matter decomposition, plant production, and multifunctionality).


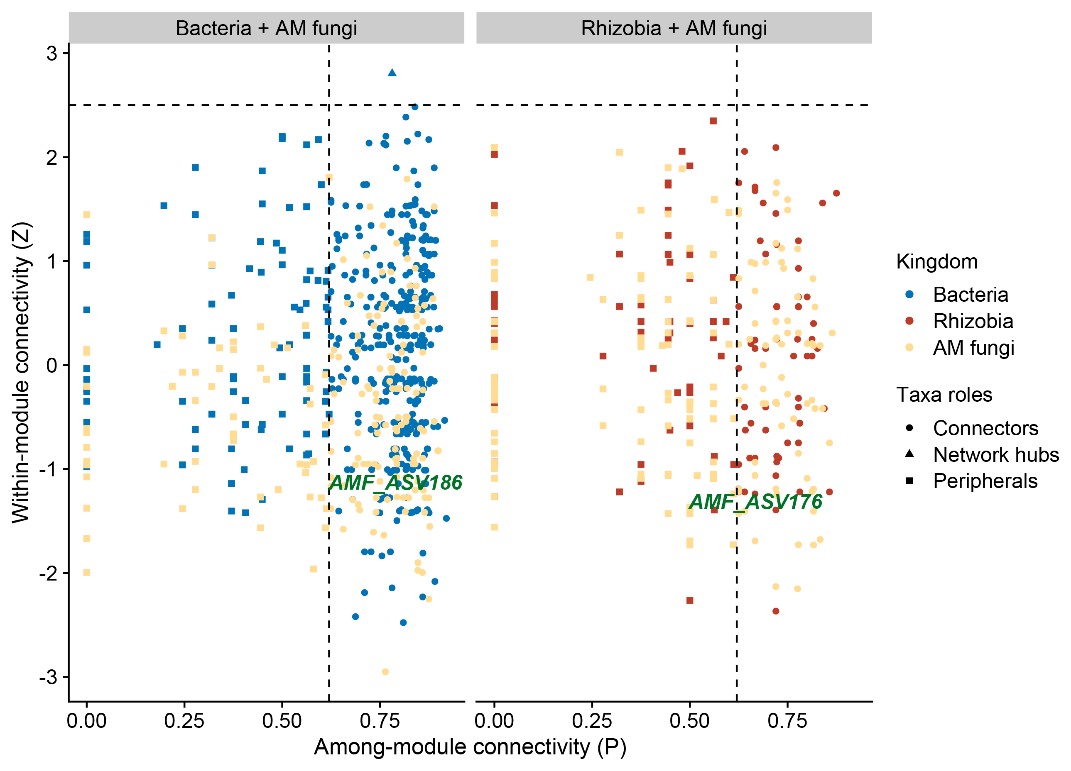


**Figure S3** Topological roles of bacterial, rhizobial, and AM fungal nodes in the cross-kingdom networks. Different colors and shapes represent the kingdoms and topological roles, respectively. Dashed lines (Z = 2.5, P = 0.62) indicate the criteria for discriminating the taxa roles. Texts in dark green denote the keystone taxa as connectors.

**
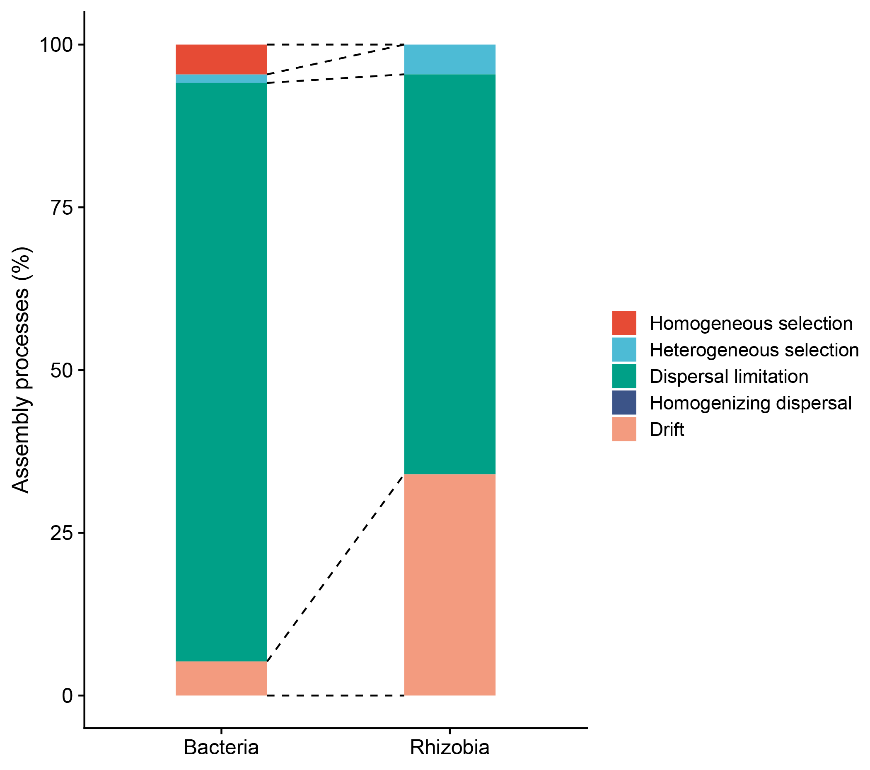
**

**Figure S4** Relative contribution of different assembly processes in shaping bacterial and rhizobial communities across the slope.

**
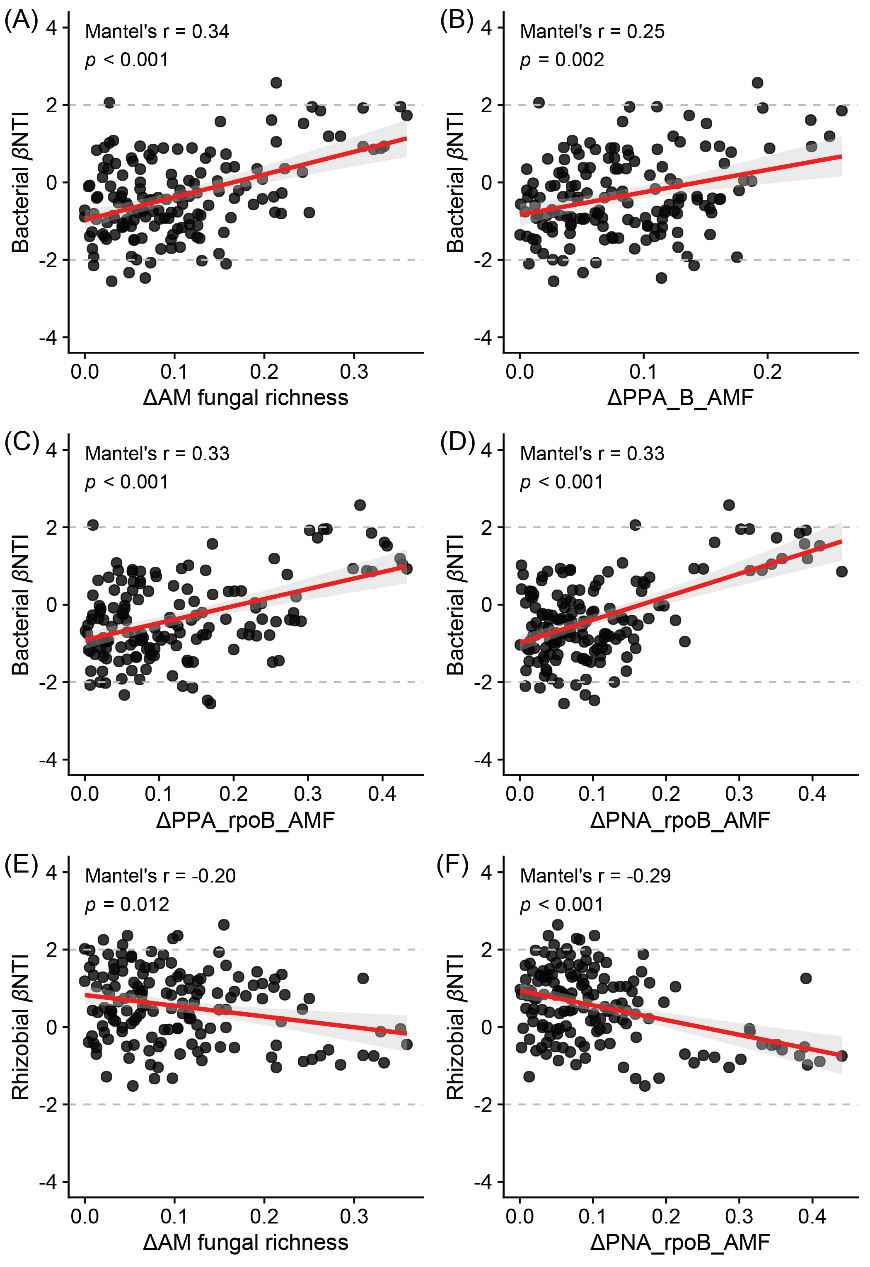
**

**Figure S5** Responses of bacterial and rhizobial *β*-nearest taxon index (*β*NTI) to changes in AM fungal richness and cross-kingdom biotic associations tested by Mantel’s tests. Red solid lines and grey shades indicate significant relationships and their 95% confidence intervals, respectively. Horizontal dashed lines denote the *β*NTI thresholds of −2 and 2. Dissimilarity matrices of biotic factors are based on Bray–Curtis distances. Δ, changes in changes in AM fungal richness or cross-kingdom biotic associations; PPA_B_AMF, putative positive association between bacteria and AM fungi; PNA_rpoB_AMF and PPA_rpoB_AMF, putative negative and positive associations between rhizobia and AM fungi, respectively.


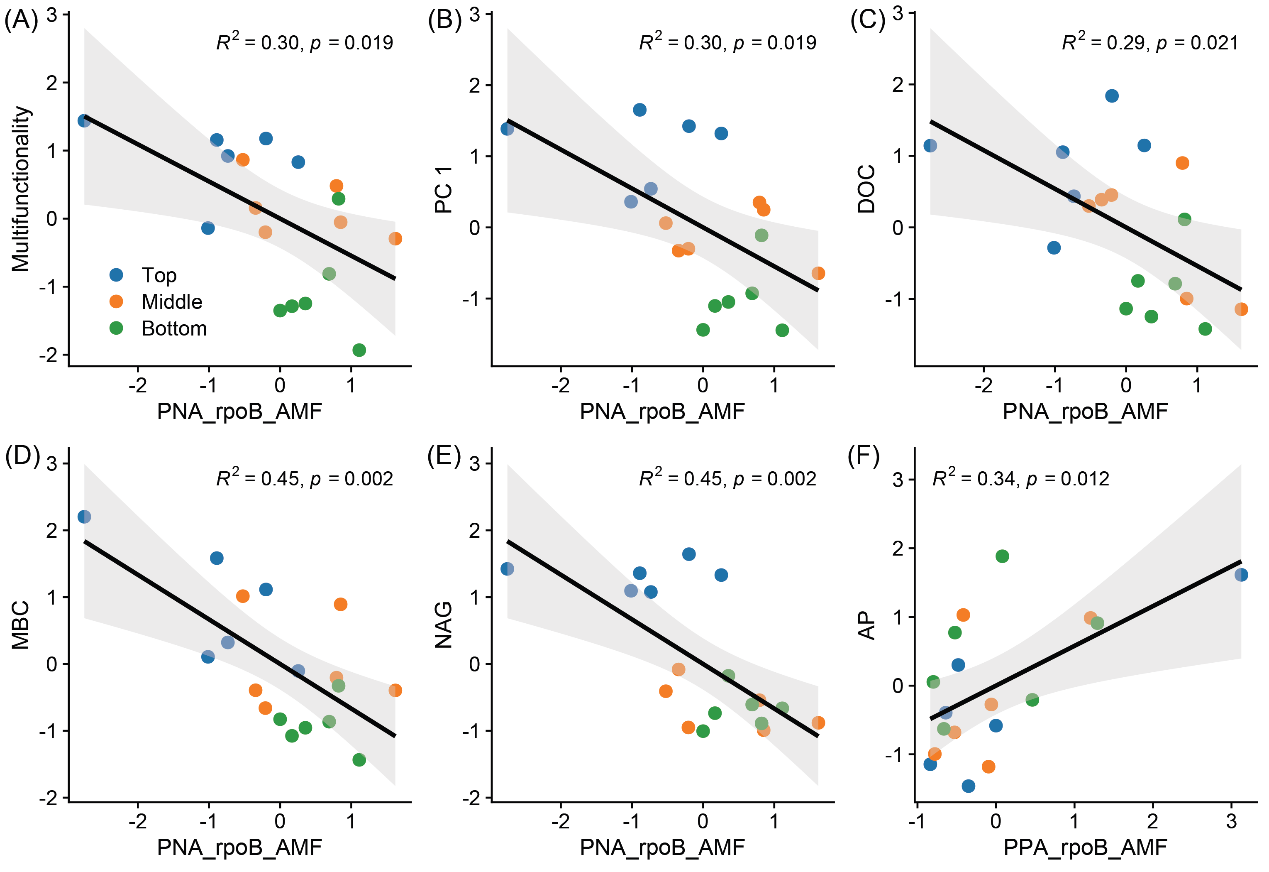


**Figure S6** Putative biotic associations between rhizobia and AM fungi driving slope multifunctionality. AP, alkaline phosphatase; DOC, dissolved organic carbon; MBC, microbial biomass carbon; NAG, N-acetylglucosaminidase; PC 1, the first axe of principal component analysis; PNA_rpoB_AMF and PPA_rpoB_AMF, putative negative and positive associations between rhizobia and AM fungi, respectively.

**
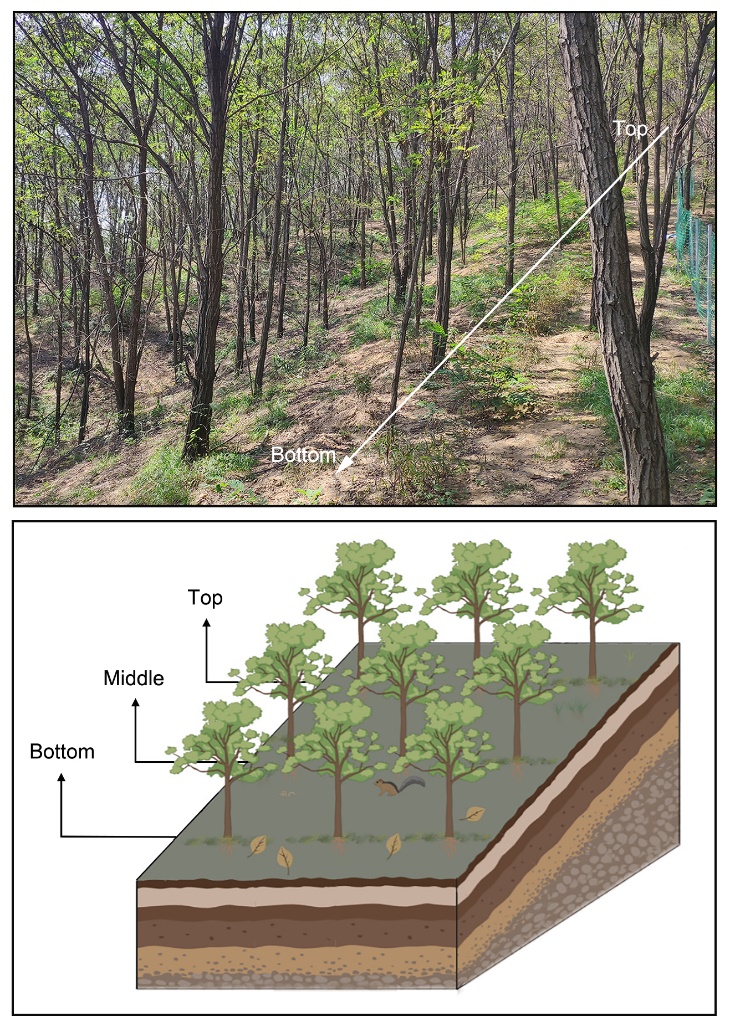
**

**Figure S7** Experimental design diagram on an eroded slope of a *Robinia pseudoacacia* plantation.
